# Cardioprotective Effect of circ_SMG6 Knockdown against Myocardial Ischemia/Reperfusion Injury Correlates with miR-138-5p-Mediated EGR1/TLR4/TRIF Inactivation

**DOI:** 10.1155/2022/1927260

**Published:** 2022-01-27

**Authors:** Chen Huang, Yalun Qu, Fan Feng, Hualu Zhang, Liliang Shu, Xiaohua Zhu, Gongcheng Huang, Jing Xu

**Affiliations:** ^1^Department of Cardiovascular Surgery, The First Affiliated Hospital of Zhengzhou University, Zhengzhou 450000, China; ^2^Department of Physiology and Pathophysiology, Tianjin Medical University, Tianjin 300070, China

## Abstract

Increased neutrophil recruitment represents a hallmark event in myocardial ischemia/reperfusion (I/R) injury due to the ensuing inflammatory response. Circular RNAs (circRNAs) are important regulatory molecules involved in cell physiology and pathology. Herein, we analyzed the role of a novel circRNA circ_SMG6 in the regulation of neutrophil recruitment following I/R injury, which may associate with the miR-138-5p/EGR1/TLR4/TRIF axis. Myocardial I/R injury was modeled in vivo by ligation of the left anterior descending (LAD) artery followed by reperfusion in mice and in vitro by exposing a cardiomyocyte cell line (HL-1) to hypoxia/reoxygenation (H/R). Gain- and loss-of-function experiments were performed to evaluate the effect of the circ_SMG6/miR-138-5p/EGR1/TLR4/TRIF axis on cardiac functions, myocardial infarction, myocardial enzyme levels, cardiomyocyte activities, and neutrophil recruitment. We found that the EGR1 expression was increased in myocardial tissues of I/R mice. Knockdown of EGR1 was found to attenuate I/R-induced cardiac dysfunction and infarction area, pathological damage, and cardiomyocyte apoptosis. Mechanistic investigations showed that circ_SMG6 competitively bound to miR-138-5p and consequently led to upregulation of EGR1, thus facilitating myocardial I/R injury in mice and H/R-induced cell injury. Additionally, ectopic EGR1 expression augmented neutrophil recruitment and exacerbated the ensuing I/R injury, which was related to the activated TLR4/TRIF signaling pathway. Overall, our findings suggest that circ_SMG6 may deteriorate myocardial I/R injury by promoting neutrophil recruitment via the miR-138-5p/EGR1/TLR4/TRIF signaling. This pathway may represent a potential therapeutic target in the management of myocardial I/R injury.

## 1. Introduction

When acute myocardial infarction occurs, blood flow exhibits immediate restoration to the ischemic myocardium, resulting in cardiomyocyte death, known as myocardial ischemia/reperfusion (I/R) injury [1]. Myocardial I/R injury is a cardiovascular disease, the deadliest disease across the globe [2]. Molecular, cellular, and tissue alterations, including cell death, inflammation, and oxidative stress, have been highlighted to play a paramount role in myocardial I/R injury, and mechanisms of myocardial I/R injury to the coronary circulation have been highlighted in cardioprotection [3, 4]. Finding possible molecular targets for myocardial I/R injury would thus be of clinical significance and might guide the search for novel treatment for this disease.

Circular RNAs (circRNAs), endogenous transcripts expressed across species, developmental stages, and pathologies, have been implicated in multiple cardiovascular diseases, including I/R injury and myocardial infarction, rendering them the potential as therapeutic targets in these diseases [5]. Such a circRNA, circ_HIPK3 has been reported to induce apoptosis of cardiomyocytes and aggravates myocardial I/R injury [6]. This study is aimed at identifying the potential role of a novel circRNA circ_SMG6 in myocardial I/R injury. The RNA22 database predicted the presence of binding sites between circ_SMG6 and microRNA (miR)-138-5p in human and mice. The overexpression of miR-138 can reduce myocardial I/R injury-induced infarct size and myocardial enzyme levels, as well as inhibiting the expression of proteins related to I/R-induced mitochondrial apoptosis by targeting HIF1-*α* [7]. Furthermore, miR-138-5p shows cardioprotective effects on cardiomyocytes against H/R-induced myocardial injury [8].

Early growth response 1 (EGR1) was predicted to be a target of miR-138-5p in the preexperiments by the RNA22 database. EGR1 is an immediate-early gene product and prototypic zinc finger transcription factor and plays critical regulatory roles in the pathobiology of multiple cardiovascular diseases [9]. Previous data has revealed that downregulation of the EGR1 expression confers protective effects on cardiomyocytes against I/R injury [10]. Also, a recent study has revealed that inhibition of EGR1 attenuates coronary microembolization-induced myocardial injury by reducing cardiomyocyte apoptosis [11]. Moreover, suppressed EGR1 expression has been confirmed to be responsible for the reduced expression of toll-like report 4 (TLR4) [12]. TLR4 is a major driver of the recruitment of neutrophils [13, 14], which are a type of inflammatory cells and recruited very early to the injured myocardium where they cause tissue injury [15]. Notably, the TLR4/TRIF signaling pathway has been documented to promote neutrophil recruitment and initiates the subsequent I/R injury [16]. Therefore, we sought to elucidate the potential role of circ_SMG6 through regulation of the miR-138-5p/EGR1/TLR4/TRIF signaling axis, in neutrophil recruitment during myocardial I/R injury.

## 2. Materials and Methods

### 2.1. Ethics Statement

The current study was approved by the Ethics Committee of the First Affiliated Hospital of Zhengzhou University and performed in strict accordance with the Guide for the Care and Use of Laboratory Animals published by the US National Institutes of Health.

### 2.2. Database Search

Myocardial I/R injury-related datasets GSE4105 and GSE67308 were retrieved from the GEO database. GSE4105 contained 6 normal samples and 6 disease samples while GSE67308 contained 4 normal samples and 4 disease samples. Due to the large difference in the expression value of samples in the GSE4105 dataset, the data were processed by log2. Differential analysis was conducted using *R* language “limma” package with │logFC > 0.7│ and *p* < 0.05 set as the threshold. Next, a protein-protein interaction (PPI) network of the screened differentially expressed genes was constructed using the STRING database, after which Cytoscape was used to plot and calculate the core degree of genes, with the top 10 with higher core degree selected. In combination with the existing literature, the key genes were determined. The miRNA mature body sequence was obtained from the miRBase database, mRNA sequence from the NCBI database, promoter sequence from the UCSC database, and circRNA sequence from the circBank database. Subsequently, the RNA22 database was applied for prediction of the miRNA-mRNA and miRNA-circRNA binding sites. Finally, hTFtarget was used to predict the transcription binding site of transcription factor and downstream gene promoter.

### 2.3. Establishment of Mouse Myocardial I/R Injury Models and In Vivo Experimental Protocols

A total of 120 healthy male C57BL/6 mice (aged 6-8 weeks, weighing 20-30 g) were purchased from Vital River Laboratory Animal Technology Co., Ltd. (Beijing, China). After one week of acclimatization, the mice were subjected to I/R mouse model establishment. Briefly, the mice were anesthetized by intraperitoneal injection of pentobarbital sodium at a dose of 40 mg/kg for general anesthesia, with the thoracic cavity exposed. Next, the left anterior descending artery (LAD) was ligated with a silk suture 6-0 using slipknot technique. Successful myocardial ischemia was observed in the electrocardiogram, as evidenced by the faded color of the myocardium, weakened pulse and ST segment elevation. After 30 min of ischemia induction, the ligature was untied to restore blood flow, and the mice were perfused for 120 min. Finally, the thoracic cavity was sutured. The mice receiving sham operation was treated the same as that of model mice except for the LAD ligation and injected with normal saline via tail vein.

Sham-operated mice served as the control and the mice following successful I/R model establishment were grouped according to presurgery injection of different plasmids carrying short hairpin RNA-negative control (sh-NC), sh-EGR1, antagomir-NC, sh-circ_SMG6, sh-circ_SMG6 + antagomir-miR-138-5p, agomir-NC, agomir-miR-138-5p, agomir-miR-138-5p + overexpression (oe)-EGR1, resatorvid (a TLR4/TRIF inhibitor), or sh-EGR1 + oe-TLR4. The presurgery injection was performed via tail vein injection at a dose of 50 nM. Sequences and plasmids used in the study were commercially synthesized (GenePharma, Shanghai, China). Each group included 8 mice (*n* = 8) for subsequent experiments. The experimental design was summarized in Supplementary Figure 1.

### 2.4. Cardiac Function Determination

The right carotid artery of mice was exposed by cervical dissection, through which, a catheter was inserted into the left ventricle. RM6240B multichannel physiological signal acquisition and processing system was connected to record the left ventricular developed pressure (LVDP), left ventricular systolic pressure (LVSP), the maximal rate of rise of the left ventricular pressure (+dp/dtmax), and the maximal rate of fall of the left ventricular pressure (-dp/dtmax).

### 2.5. Electrocardiogram

Two-dimensional electrocardiogram was performed with an electrocardiogram machine (GE Vivid 7.0; General Electric Company, Schenectady, NY) and a 20 MHz probe. All measured values represent the average of five consecutive cardiac cycles. Left ventricular ejection fraction (LVEF) and left ventricular fractional shortening (LVFS) were automatically calculated by computer algorithms.

### 2.6. TTC Staining

After reperfusion, the mouse heart was quickly removed and stored at -80°C for 30 min. The myocardium was cut into 2 mm-thick sections, which were incubated with 1% TTC solution in the dark at 37°C for 15 min, and fixed in 10% formaldehyde solution. The sections were photographed, and the ImageJ software) was used to quantify the infarct area. The ratio of the infarct area to the ischemic area represents the infarct area ratio.

### 2.7. Lactic Acid Dehydrogenase (LDH) and Creatine Kinase (CK) Activity Determination

After I/R modeling, the femoral artery blood was removed from the mice, centrifuged at 1000 r/min and 4°C for 15 min to separate serum, which was then stored at -80°C. LDH kit (C0017, Beyotime Biotechnology, Shanghai, China) and CK MB kit (ML-ELISA-0252, R&D Systems) were used to determine the activity of LDH and CK in mouse serum and in the supernatant of HL-1 cells in each group.

### 2.8. Detection of Malondialdehyde (MDA) Level and Superoxide Dismutase (SOD) Activity

After establishment of I/R injury, myocardial tissues were obtained and washed with cold normal saline to remove blood. After quick freezing in liquid nitrogen, the samples were stored at -80°C. Next, the samples were cut into small pieces, centrifuged at 15000 × g for 14 min, and homogenized with normal saline. Afterwards, 0.5 mL of supernatant was collected, and the corresponding kit was used to detect the SOD activity (ab65354, Abcam Inc., Cambridge, UK) and MDA level (ab118970, Abcam) in the myocardial tissue.

### 2.9. Hematoxylin-Eosin (HE) Staining

Sections of myocardial tissues were stained with hematoxylin for 5 min. After washing, sections were immersed in 1% hydrochloric acid for 2 s, dehydrated, stained with eosin for 5 s, and dehydrated again. Following dewaxing and sealing, sections were observed under a brightfield microscope, with the nucleus stained blue and the cytoplasm stained red.

### 2.10. TUNEL Staining

The apoptosis in myocardial tissues was quantified with TUNEL staining following the protocols of the TUNEL apoptosis detection kit (11684817910, Roche, Mannheim, Germany). Paraffin-embedded sections of the myocardial tissues were prepared, dewaxed, hydrated, and treated with proteinase K for 20 min. The sections were then incubated in 30% H_2_O_2_ methanol solution for 5 min to inactivate endogenous peroxidase and treated with equilibration buffer for 30 min at room temperature. Thereafter, TUNEL reagent (Roche, Indianapolis, IN) was added to sections, incubated at 37°C for 1 h, followed by visualization with DAB reagent. The sections were then subjected to fluorescence microscopy, wherein 5 fields of view were selected in each section, and images were analyzed with the BI-2000 system to quantify TUNEL-positive cells (in pale brown).

### 2.11. circRNA Sequencing for Myocardial Tissues

Two mouse models of myocardial I/R injury were constructed for sequencing, and two normal heart samples were used as the control. These four samples were regarded as sequencing samples. Total RNA was extracted from each sample using TRIzol Reagents (Invitrogen Inc., Carlsbad, CA). The concentration, purity, and integrity of RNA were determined by Qubit®RNA analysis kit of Qubit®2.0 Fluorometer® (Life Technologies, Carlsbad, CA), nanometer spectrophotometer (IMPLEN, California), and RNA Nano 6000 analysis kit (Agilent Laboratories, Molecular Technology Laboratory, Santa Clara, CA) of the Bioanalyzer 2100 system, respectively. Total RNA (3 *μ*g) was taken as the input for RNA sample preparation. The cDNA library generated by the NEBNext®UltraTM RNA kit (Illumina, Nebraska) was used and evaluated on the Agilent 2100 Bioanalyzer system. TruSeq PE Cluster Kit v3 cBot HS (Illumina) was used to cluster the index coding samples on the cBot cluster generation system, followed by sequencing on the Illumina-Hiseq 550 platform, and 125 bp/150 bp paired end reading was generated.

### 2.12. Dual Luciferase Reporter Assay

HEK-293T cell line (Cell Bank of the Shanghai Institute of Life Science, Chinese Academy of Sciences, Shanghai, China) was cultured in DMEM supplemented with 10% FBS and 1% penicillin-streptomycin. Upon 80-90% confluence, cells were trypsinized, passaged, and cultured (5% CO_2_, 37°C). The gene targeted by miR-138-5p was predicted with the Targetscan database and validated utilizing the dual luciferase reporter assay. EGR1-3′-untranslated region (3′-UTR) gene fragment was synthesized artificially and introduced into the pmirGLO vector (Promega Corporation, Madison, WI) using endonuclease sites. The complementary sequence mutation site (CGTACTG) of the seed sequence was designed on the EGR1 wild-type (WT), after which the target fragment was inserted into the pGL3-control vector using T4 DNA ligase via restriction endonuclease cleavage. Next, the luciferase reporter plasmids EGR1-WT and EGR1-MUT were cotransfected with mimic-NC or miR-138-5p mimic into HEK-293T cells. After 48 h of transfection, cells were collected and lysed. The luciferase activity was detected using the Dual-Luciferase Reporter Assay System (Promega) on a TD 20/20 luminometer (E5311, Promega).

### 2.13. Hypoxia/Reoxygenation (H/R) Cell Model Construction and In Vitro Experimental Protocols

A mouse cardiomyocyte cell line HL-1 was purchased from Wuhan Procell Life Science & Technology (Wuhan, China). Stably transfected cells were cultured in serum-free DMEM at 37°C under 5% CO_2_, 1% O_2_, and 94% N_2_ for 24 h and then under 5% CO_2_ and 95% O_2_ at 37°C for 3 h to induce cell injury through H/R. Next, the medium was replaced with complete DMEM medium.

With untreated cells (the normal group) served as the control, H/R-treated cells fell into groups corresponding to different transections (sh-NC, sh-circ_SMG6, sh-circ_SMG6 + miR-138-5p inhibitor, NC mimic, miR-138-5p mimic, or miR-138-5p mimic + oe-EGR1) before the H/R, except for the H/R group wherein cells received no treatment before H/R. Meanwhile, there were other cells treated with NC mimic, miR-138-5p-mimic, NC inhibitor, or miR-138-5p-inhibitor in the absence of H/R. Specifically, cell transfection with corresponding plasmids (50 ng) was performed according to protocols of the Lipofectamine 2000 reagent (Invitrogen). Experimental design was summarized in Supplementary Figure 1.

### 2.14. RNA Binding Protein Immunoprecipitation (RIP)

RIP assay was performed according to the protocol of the EZ-Magna RIP kit (Millipore, Billerica, MA). HL-1 cells with biotin-NC and biotin-miR-138-5p were lysed in complete RIP lysis buffer. An equal volume of cell extract (100 *μ*L) was incubated with mouse anti-Ago2 conjugated magnetic beads in RIP buffer. The cell extract incubated with normal mouse IgG served as NC. Finally, RNA was extracted by the phenol-chloroform method and quantified by qRT-PCR.

### 2.15. Flow Cytometry

Cells were trypsinized, centrifuged (1000 × g, 5 min), and resuspended in 100 *μ*L PBS, followed by 15-min incubation at room temperature in the dark with 5 *μ*L annexin V-fluorescein isothiocyanate (FITC) and 10 *μ*L propidium iodide (PI). A flow cytometer (FACS Calibur; Becton Dickinson, San Jose, CA) was applied to measure the apoptosis rate of the cell suspension.

### 2.16. MTT Assay

HL-1 cells were seeded into a 48-well plate (1 × 10^4^ cells per well) preheated to 37°C, with 200 *μ*L of DMEM-diluted MTT reagent added to each well to incubate in the dark at 37°C for 4 h. Thereafter, the MTT reagent was replaced by 200 *μ*L DMSO, and the cells continued to incubate on a shaker in the dark for 20 min, whereupon the optical density (OD) value at 490 nm was measured with a microplate reader. Cell viability was calculated as the ratio of the OD value of the test sample to that of the normal sample.

### 2.17. Two-Photon Microscope

Time-lapse imaging was performed using a customized two-photon microscope with ImageWarp version 2.1 acquisition software (A&B software). The recipient mice were anesthetized by intraperitoneal injection of ketamine (50 mg/kg) and xylazine (10 mg/kg), and the dose was halved every hour. The mouse was intubated through the mouth and trachea with a No. 20 vascular catheter and ventilated with moisture of 120 breaths per minute and 0.5 mL of indoor air. For time-lapse imaging of neutrophil migration, we obtained an average of 15 video rate frames (0.5 s per piece) during the acquisition process to match the ventilator rate and minimize motion artifacts. Each plane represents an image size of 220 × 240 *μ*m in *x* and *y*. A total of 21 continuous plane dimensions (2.5 *μ*m each) were acquired in *z* to form a *Z* stack. The neutrophils were tracked from their first appearance in the imaging window until they shifted more than 20 *μ*m from the starting position. The percentage of exudative neutrophils was expressed as the ratio of number of extravascular neutrophils to the sum of intravascular and extravascular neutrophils. In 50 *μ*L PBS, 15 *μ*L 655 nm nontargeted *Q*-spot was injected intravenously to visualize blood vessels. Two-photon excitation generated a second harmonic signal from the collagen in the myocardial tissues. Imaris (Bitplane) was used to complete multidimensional rendering and manual unit tracking. Data were transferred and plotted in GraphPad Prism 6.0 (Sun Microsystems Inc.) to create the graph.

### 2.18. RNA Isolation and Quantitation

miRNA was isolated from EVs, tissues, and cells using the mirVanaTM PARISTM RNA kit (AM1556, Invitrogen). For mRNA analysis, First Strand cDNA Synthesis Kit (K1622, Fermentas Inc., Hanover, MD) was used to randomly synthesize cDNA from 1 *μ*g of total RNA. For miRNA analysis, TaqMan microRNA Reverse Transcription Kit (4366597, Applied Biosystems Inc. Carlsbad, CA) was used to synthesize cDNA of miRNA. Next, RNA was quantitatively analyzed using Fast SYBR Green PCR kit (Applied Biosystems) and ABI PRISM 7300 RT-PCR system (Applied Biosystems), with three repeated wells for each sample. U6 served as the internal reference for miR-138-5p, while GAPDH for the remaining genes. All primers were purchased from Sangon Biotechnology (Shanghai, China), and the sequences are shown in Supplementary Table 1. Besides, reverse primers of TaqManTM microRNA Reverse Transcription Kit were used.

### 2.19. Western Blot Analysis

Total protein was extracted from cells with 60 *μ*L RIPA lysis buffer containing 1% protease inhibitor and 1% phosphorylase inhibitor (Beyotime). The protein concentration was then determined with a bicinchoninic acid kit (Beyotime). After separation by 10% sodium dodecyl sulfate polyacrylamide gel electrophoresis, 20 *μ*g protein was transferred onto a polyvinylidene fluoride membrane. Next, the membrane was treated with 5% BSA at room temperature for 2 h and underwent overnight incubation at 4°C with primary antibodies (Supplementary Table 2), followed by 1-h incubation with horseradish peroxidase-labeled secondary antibody (Supplementary Table 2) at room temperature. Afterwards, the immunocomplexes on the membrane were visualized using enhanced chemiluminescence (ECL) reagent (BM101, Biomiga Inc.) and developed using BioSpectrum 600 Imaging System (Ultra-Violet Products, Cambridge, UK) after which the gray value of protein bands was quantified by ImageJ software.

### 2.20. Statistical Analysis

All data were processed using SPSS 21.0 statistical software (IBM Corp. Armonk, NY) and GraphPad Prism 7 (GraphPad Software, La Jolla, CA). Measurement data were expressed as mean ± standard deviation. Data between two groups were analyzed using the unpaired *t*-test while those among multiple groups were analyzed by one-way analysis of variance (ANOVA). *p* < 0.05 was considered as statistically significant.

## 3. Results

### 3.1. EGR1 Is Highly Expressed in Myocardial Tissues of Surgically Modeled Mice with Myocardial I/R Injury

**Figure 1:**
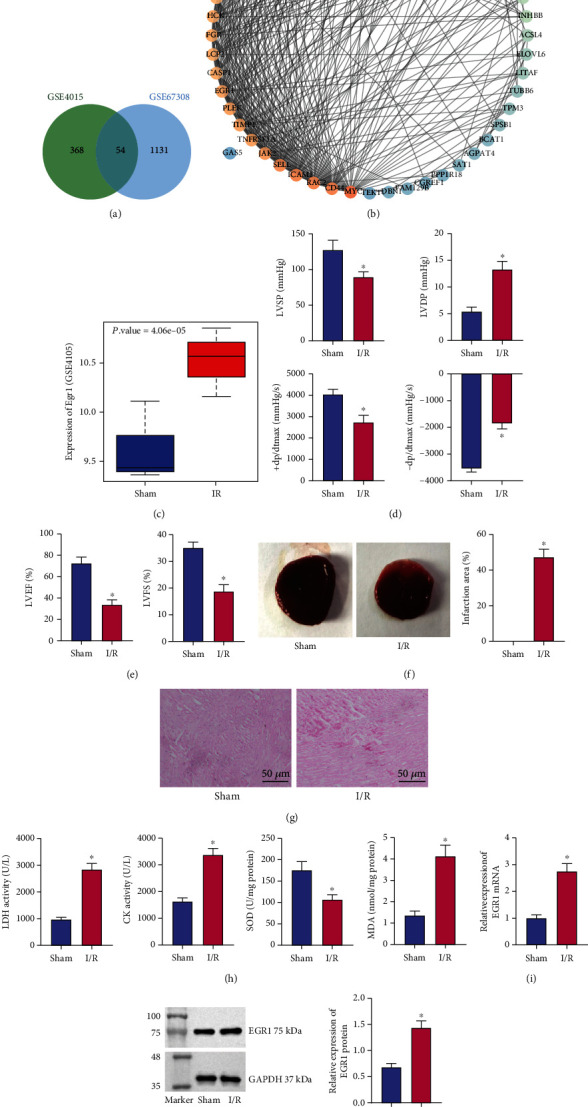
Bioinformatic analysis of I/R injury-related genes and upregulated expression of EGR1 in myocardial tissues of I/R mice. (a) Venn diagram analysis of the significantly upregulated genes in myocardial tissues of I/R mice in the GSE4105 and GSE67308 datasets. (b) A PPI network of the 54 intersected genes. The redder color of the circle where the gene is reflects the higher core degree, and the bluer color indicates lower core degree. (c) A box plot of the expression data of EGR1 in normal (*n* = 6) and disease samples (*n* = 6) in the GSE4105 dataset. (d) Detection of LVSP, LVDP, +dp/dtmax, and -dp/dtmax related to cardiac function in I/R and sham-operated mice. (e) LVFS% and LVEF% detected by electrocardiogram in I/R and sham-operated mice. (f) TTC staining of myocardial tissues of I/R and sham-operated mice. (g) HE staining of myocardial tissues of I/R and sham-operated mice. (h) LDH and CK levels in arterial blood and MDA and SOD levels in myocardial tissues in I/R and sham-operated mice. (i) EGR1 mRNA expression determined by qRT-PCR in myocardial tissues of I/R and sham-operated mice. (j) EGR1 protein expression determined by Western blot analysis in myocardial tissues of I/R and sham-operated mice. ^∗^*p* < 0.05 vs. the sham-operated mice or control cells. *n* = 8 for mice following each treatment.

Differential analysis on normal samples and I/R injury samples from GSE4105 and GSE67308 datasets revealed 422 and 1185 significantly upregulated genes in myocardial tissues with I/R injury, respectively. Following intersection analysis of these genes from the two datasets, 54 genes were identified (Figure 1(a)). A PPI network involving the 54 genes was constructed, and the top 10 core genes were extracted (Figure 1(b), Supplementary Table 3). Among these 10 genes, inhibition of the expression of EGR1 gene has been reported to attenuate myocardial damage [11, 17], and meanwhile, EGR1 was found to be highly expressed in myocardial I/R samples in the GSE4105 dataset (Figure 1(c)). Therefore, EGR1 was selected as the target gene for follow-up research.

As shown in Figures 1(d) and 1(e), the results of cardiac function detection and electrocardiogram presented higher LVDP yet lower LVSP, +dp/dtmax, -dp/dtmax, LVFS%, and LVEF% in the surgically modeled mice relative to sham-operated mice. TTC staining indicated increased infarct size in I/R mice (Figure 1(e)). Additionally, HE staining data showed significant myocardial hemorrhage, myocardial structural changes, and neutrophil infiltration in surgically modeled mice (Figure 1(g)). Versus sham-operated mice, surgically modeled mice exhibited upregulated LDH and CK activities in arterial blood and MDA level in myocardial tissues yet reduced SOD activity in myocardial tissues (Figure 1(h)). Furthermore, the results of qRT-PCR and Western blot analyses demonstrated an increase of the EGR1 expression in the myocardial tissues of surgically modeled mice relative to sham-operated mice (Figure 1(i) and 1(j)).

These results collectively validated the successful establishment of myocardial I/R injury in the surgically modeled mice (hereinafter referred to as I/R mice), in which the overexpression of EGR1 occurred.

### 3.2. EGR1 Silencing Reduces Myocardial Injury in I/R Mice

**Figure 2:**
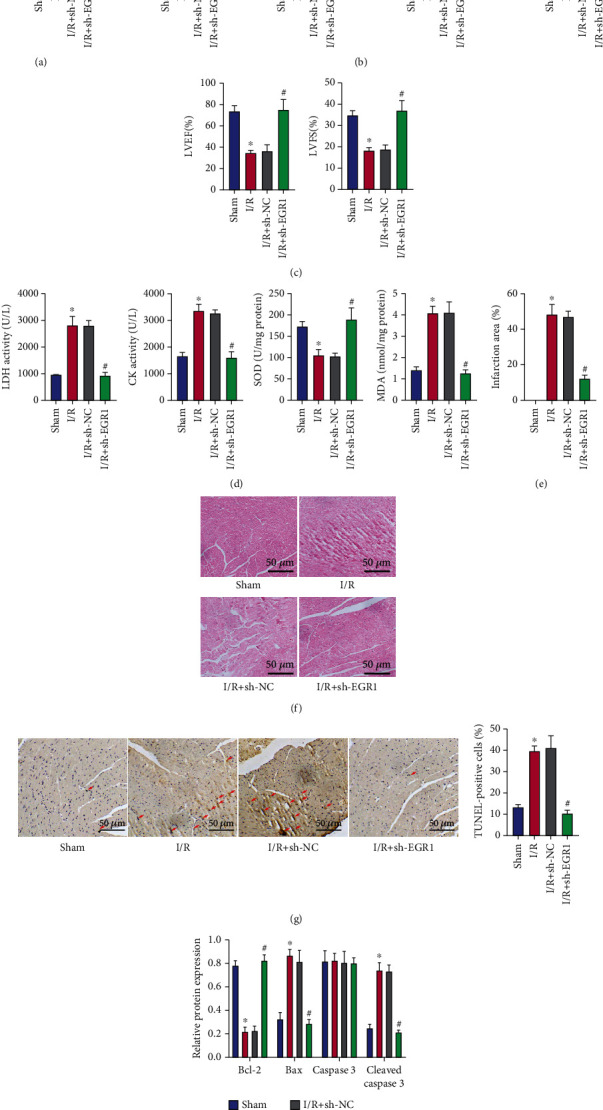
Knockdown of EGR1 attenuates myocardial injury in I/R mice. (a) Silencing efficiency of sh-EGR1 confirmed by qRT-PCR in myocardial tissues of sham-operated mice or I/R mice in the presence/absence of sh-EGR1 treatment. (b) Detection of LVSP, LVDP, +dp/dtmax, and -dp/dtmax related to cardiac function in sham-operated mice or I/R mice in the presence/absence of sh-EGR1 treatment. (c) LVFS% and LVEF% detected by electrocardiogram in sham-operated mice or I/R mice in the presence/absence of sh-EGR1 treatment. (d) Activity LDH and CK in the arterial blood and SOD and MDA levels in the myocardial tissues of sham-operated mice or I/R mice in the presence/absence of sh-EGR1 treatment. (e) TTC staining of myocardial tissues of sham-operated mice or I/R mice in the presence/absence of sh-EGR1 treatment. (f) HE staining of myocardial tissues of sham-operated mice or I/R mice in the presence/absence of sh-EGR1 treatment. (g) Apoptosis of cardiomyocytes in myocardial tissues of sham-operated mice or I/R mice in the presence/absence of sh-EGR1 treatment, as assessed by TUNEL assay. (h) Protein expression of Bax, cleaved caspase-3, and Bcl-2 determined by Western blot analysis in myocardial tissues of sham-operated mice or I/R mice in the presence/absence of sh-EGR1 treatment. ^∗^*p* < 0.05 vs. the sham-operated mice. #*p* < 0.05 vs. I/R mice treated with sh-NC. *n* = 8 for mice following each treatment.

We then moved to verify the potential role of EGR1 in myocardial I/R injury. qRT-PCR results confirmed the silencing efficiency of sh-EGR1, as shown by the decreased expression of EGR1 in the myocardial tissues of I/R mice treated with sh-EGR1 (Figure 2(a)).

Moreover, EGR1 silencing was observed to reverse the promoting effect of I/R on LVDP and the inhibitory effect on LVSP, +dp/dtmax, -dp/dtmax, LVFS%, and LVEF% (Figures 2(b) and 2(c)). In addition, knockdown of EGR1 in I/R mice resulted in downregulated LDH and CK levels in arterial blood as well as a reduced MDA level and an elevated SOD level in myocardial tissues (Figure 2(d)).

Analysis on the mouse myocardial tissues using TTC and HE staining suggested reduced necrotic myocardium, myocardial tissue bleeding, and neutrophil infiltration following knockdown of EGR1, and muscle structure was restored (Figures 2(e) and 2(f)).

Meanwhile, I/R mice, relative to sham-operated mice, presented with higher apoptosis rate of cardiomyocytes, accompanied by elevated protein expression of proapoptotic Bax and cleaved caspase-3 and reduced protein expression of antiapoptotic Bcl-2 in myocardial tissues, whereas these effects of I/R were abolished in the absence of EGR1 (Figures 2(g) and 2(h)).

The aforementioned results showed that knockdown of EGR1 could reduce I/R-induced cardiac dysfunction, area of myocardial infarction, and pathological damage of myocardial tissues and cardiomyocyte apoptosis.

### 3.3. circ_SMG6 Upregulates the Expression of EGR1 by Competitively Binding to miR-138-5p

**Figure 3:**
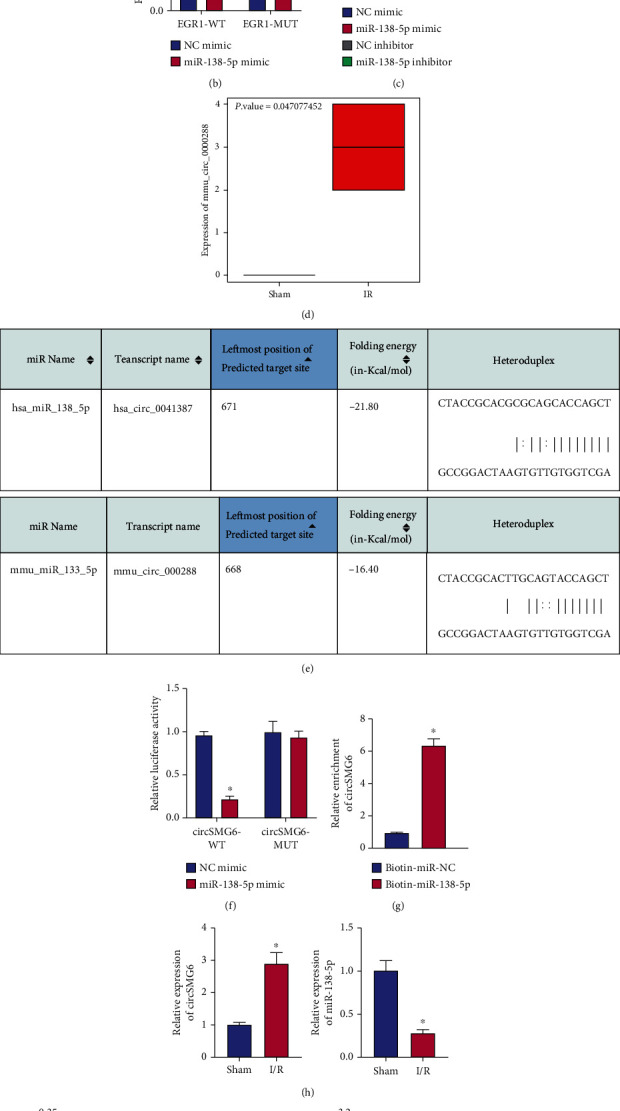
circ_SMG6 competitively binds to miR-138-5p and thus attenuates the binding of miR-138-5p to EGR1. (a) Binding sites between miR-138-5p and EGR1 in human (the top) and mice (the bottom) predicted by the RNA22 database. (b) Binding of miR-138-5p to EGR1 verified by dual-luciferase reporter assay in HEK-293 T cells. (c) Expression of miR-138-5p and EGR1 determined with qRT-PCR in HL-1 cells treated with miR-138-5p mimic/inhibitor. (d) A box plot of the mmu_circ_0000288 (circ_SMG6) expression in myocardial samples of I/R mice (*n* = 2) and sham-operated mice (*n* = 2) in circRNA sequencing. (e) Binding sites between miR-138-5p and circ_SMG6 in human and mice predicted by the RNA22 database. (f) Binding of miR-138-5p to circ_SMG6 verified by dual-luciferase reporter assay in HEK-293T cells. (g) Enrichment of circ_SMG6 determined by RIP assay in the cells treated with biotin-miR-138-5p. (h) Expression of miR-138-5p and circ_SMG6 determined by qRT-PCR in myocardial tissues of I/R and sham-operated mice. (i) Correlation of circ_SMG6 expression with the miR-138-5p expression and EGR1 expression analyzed by Pearson's correlation coefficient in myocardial tissues of I/R mice. (j) Expression of circ_SMG6, miR-138-5p, and EGR1 determined with qRT-PCR in HL-1 cells treated with oe-circ_SMG6 or sh-EGR1 or their combination. ^∗^*p* < 0.05 vs. the sham-operated mice, HEK-293T cells transfected with NC mimic or HL-1 cells treated with biotin-NC or HL-1 cells treated with oe-NC + sh-NC; #*p* < 0.05 vs. cells treated with oe-circ_SMG6 + sh-NC. Cell experiments were conducted three times independently. *n* = 8 for mice following each treatment.

Next, we aimed to exploit the upstream regulatory mechanism of EGR1. The RNA22 database predicted that EGR1 was targeted by miR-138-5p in both human and mice (Figure 3(a)). Dual-luciferase reporter assay data further showed that the miR-138-5p overexpression in HEK-293T cells could inhibit the luciferase activity of EGR1-3′-UTR-WT, but had no effect on that of EGR1-3′-UTR-MUT (Figure 3(b)). These data validated the binding affinity between miR-138-5p and EGR1.

Further to define the effect of miR-138-5p on the EGR1 expression, we manipulated miR-138-5p expression in HL-1 cells. The mRNA expression of EGR1 was displayed to be diminished in the presence of the elevated expression of miR-138-5p induced by miR-138-5p mimic, and miR-138-5p inhibitor-mediated downregulation of miR-138-5p co-occurred with an increase in the EGR1 expression (Figure 3(c)). It can thus be assumed that miR-138-5p inversely regulated the EGR1 expression.

The results of circRNA sequencing used for determining the upstream factor of miR-138-5p illustrated that the mmu_circ_0000288 (circ_SMG6) overexpression occurred in the myocardial tissues of I/R mice (Figure 3(d)), suggesting that mmu_circ_0000288 may promote I/R injury. The RNA22 database predicted that the sequence of circ_SMG6 (hsa_circ_0041387, mmu_circ_0000288) in human and mice was conserved, and that only circ_SMG6 was targeted by miR-138-5p in both human and mice (Figure 3(e)). Further, the miR-138-5p overexpression in HEK-293T cells could decrease the luciferase activity of circ_SMG6-3′-UTR-WT without affecting that of circ_SMG6-3′-UTR-MUT (Figure 3(f)). RIP data exhibited that the enrichment of circ_SMG6 was enhanced in the HL-1 cells treated with biotin-miR-138-5p (Figure 3(g)). The above results indicated a direct interaction between circ_SMG6 and miR-138-5p.

Further detection with qRT-PCR showed an increase in the circ_SMG6 expression and a decline of the miR-138-5p expression in the myocardial tissues of I/R mice compared with the sham-operated mice (Figure 3(h)). Pearson's correlation coefficient presented that the circ_SMG6 expression was negatively correlated with the miR-138-5p expression while positively correlated with the EGR1 expression in myocardial tissues of I/R mice (Figure 3(i)). Overall, these findings suggested that circ_SMG6 may promote the EGR1 expression by competitively inhibiting miR-138-5p.

Further to examine this speculation, we overexpressed circ_SMG6 in the presence/absence of simultaneous downregulation of EGR1 in HL-1 cells. As depicted in Figure 3(j), oe-circ_SMG6 treatment alone led to elevated levels of circ_SMG6 and EGR1 and reduced miR-138-5p levels, whereas its combination with sh-EGR1 reversed the increase in EGR1 expression yet had no influence on circ_SMG6 and miR-138-5p expression. Meanwhile, sh-EGR1 itself only downregulated the EGR1 expression without obvious effects on circ_SMG6 and miR-138-5p expression (Figure 3(j)).

These results collectively indicated that circ_SMG6 negatively mediated miR-138-5p and thus promoted EGR1 expression, through which the circ_SMG6/miR-138-5p/EGR1 axis confers a role in I/R injury.

### 3.4. circ_SMG6 Silencing Alleviates Myocardial I/R Injury by Enhancing miR-138-5p-Mediated EGR1 Inhibition In Vivo

**Figure 4:**
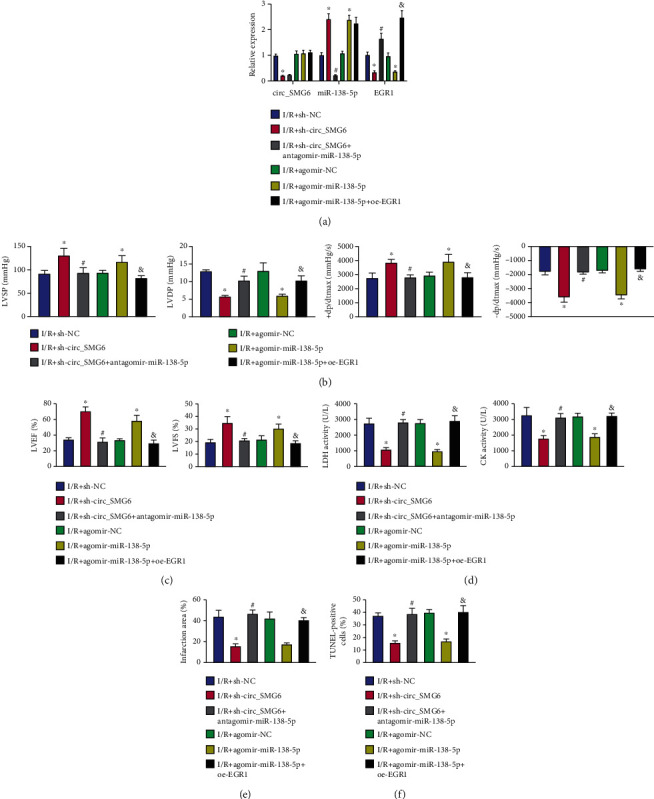
Knockdown of circ_SMG6 alleviates myocardial I/R injury by upregulating miR-138-5p and reducing EGR1 expression in vivo. I/R mice were treated with sh-circ_SMG6, sh-circ_SMG6 + antagomir-miR-138-5p, agomir-miR-138-5p, or agomir-miR-138-5p + oe-EGR1. (a) Expression of circ_SMG6, miR-138-5p, and EGR1 measured by qRT-PCR in myocardial tissues of mice in each group. (b) Detection of LVSP, LVDP, +dp/dtmax, and -dp/dtmax related to cardiac function in I/R mice. (c) LVFS% and LVEF% detected by electrocardiogram in I/R mice. (d) Activity of LDH and CK in the arterial blood of I/R mice. (e) TTC staining of myocardial tissues of I/R mice. (f) Apoptosis of cardiomyocytes in myocardial tissues of I/R mice assessed by TUNEL assay. ^∗^*p* < 0.05 vs. the I/R mice treated with sh-NC or agomir-NC. #*p* < 0.05 vs. the I/R mice treated with sh-circ_SMG6. &*p* < 0.05 vs. the I/R mice treated with agomir-miR-138-5p. *n* = 8 for mice following each treatment.

We then carried out functional rescue experiments in I/R mice to further elucidate whether circ_SMG6 affects myocardial I/R injury through the miR-138-5p-mediated EGR1 inhibition. According to qRT-PCR analysis of the myocardial tissue of I/R mice following various treatments (Figure 4(a)), shRNA-mediated silencing of circ_SMG6 led to a reduced myocardial level of EGR1 yet an increased miR-138-5p level, and further silencing of miR-138-5p negated the reduction of EGR1 level but exerted no effects on the circ_SMG6 expression. Moreover, agomir targeting miR-138-5p led to the upregulated miR-138-5p expression along with the reduced EGR1 expression and almost unchanged circ_SMG6 expression, based on which additional upregulation of the EGR1 expression showed no obvious effects on circ_SMG6 and miR-138-5p expression.

Downregulation of circ_SMG6 resulted in promotion of cardiac function-related indexes LVSP, +dp/dtmax, -dp/dtmax, LVFS%, and LVEF% while LVDP was decreased (Figures 4(b) and 4(c)). In addition, there was a reduction in the LDH and CK activity in arterial blood (Figure 4(d)), myocardial infarct size (Figure 4(e)), and cardiomyocyte apoptosis (Figure 4(f)), indicating the alleviation of myocardial I/R damage. Meanwhile, combined treatment of sh-circ_SMG6 and antagomir-miR-138-5p was observed to aggravate I/R injury; whereas treatment with agomir-miR-138-5p alone in I/R mice alleviated I/R injury, which was abrogated by further overexpression of EGR1 (Figures 4(b)–4(f)).

Cumulatively, our data evidenced that circ_SMG6 knockdown elevated miR-138-5p to downregulate EGR1 expression and reduce I/R injury in vivo.

### 3.5. circ_SMG6 Silencing Restricts H/R-Induced Cardiomyocyte Injury and Apoptosis by Enhancing miR-138-5p-Mediated EGR1 Inhibition

**Figure 5:**
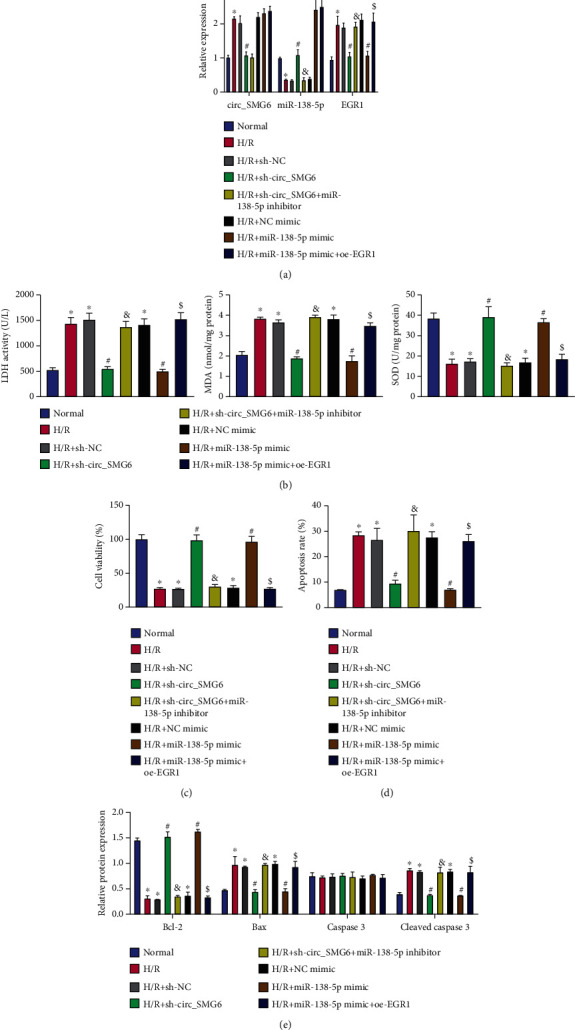
circ_SMG6 silencing impedes H/R-induced cardiomyocyte injury and apoptosis by enhancing miR-138-5p-mediated EGR1 inhibition. Normal cells served as control and H/R-induced HL-1 cells were transfected with sh-circ_SMG6, sh-circ_SMG6 + miR-138-5p inhibitor, miR-138-5p mimic, or miR-138-5p mimic + oe-EGR1. (a) Expression of circ_SMG6, miR-138-5p, and EGR1 measured by qRT-PCR in myocardial tissues of mice in each group. (b) Detection of LDH and SOD activity and MDA level in the supernatant of H/R-induced HL-1 cells. (c) HL-1 cell viability measured by MTT assay. (d) Flow cytometric analysis of HL-1 cell apoptosis rate. (e) Protein expression of apoptosis-related Bax, caspase-3, cleaved caspase-3, and Bcl-2 determined by Western blot analysis in H/R-induced HL-1 cells. ^∗^*p* < 0.05 vs. the normal cells. #*p* < 0.05 vs. the H/R-treated HL-1 cells transfected with sh-NC or NC mimic. &*p* < 0.05 vs. the H/R-treated HL-1 cells transfected with sh-circ_SMG6. $*p* < 0.05 vs. the H/R-treated HL-1 cells transfected with miR-138-5p mimic. Cell experiments were conducted three times independently.

We next investigate the effect of circ_SMG6/miR-138-5p/EGR1 coexpression network on H/R-induced cardiomyocyte injury. As measured with qRT-PCR, expression levels of circ_SMG6 and EGR1 were upregulated, and the miR-138-5p level was reduced in response to H/R; additional shRNA-mediated knockdown of circ_SMG6 led to downregulated EGR1 expression and elevated miR-138-5p expression, and further, downregulation of miR-138-5p reversed the decrease in the EGR1 expression yet not affected the circ_SMG6 expression. Moreover, miR-138-5p mimic-mediated upregulation of miR-138-5p co-occurred with the downregulated EGR1 expression and unaffected circ_SMG6 expression, based on which the simultaneous upregulation of EGR1 showed no obvious effects on circ_SMG6 and miR-138-5p expression (Figure 5(a)).

Further, H/R-treated HL-1 cells presented with elevated LDH activity and MDA level (detected in cell supernatant) and apoptosis rate, and lower SOD activity (detected in cell supernatant) and cell viability. In the absence of circ_SMG6, the aforementioned effect caused by H/R was negated, with the cardiomyocyte injury alleviated. Conversely, simultaneous silencing of circ_SMG6 and miR-138-5p could aggravate the cardiomyocyte injury and promote cell apoptosis. In response to miR-138-5p mimic, the cardiomyocyte injury and apoptosis were observed to be attenuated, which was reversed following additional overexpression of EGR1 (Figures 5(b)–5(e)).

Thus, it was reasonable to suggest that circ_SMG6 knockdown might reduce cardiomyocyte injury and apoptosis by elevating miR-138-5p-mediated EGR1 inhibition.

### 3.6. EGR1 Knockdown Reduces the Recruitment of Neutrophils and Alleviates the Ensuing Myocardial I/R Injury by Inactivating the TLR4/TRIF Signaling

**Figure 6:**
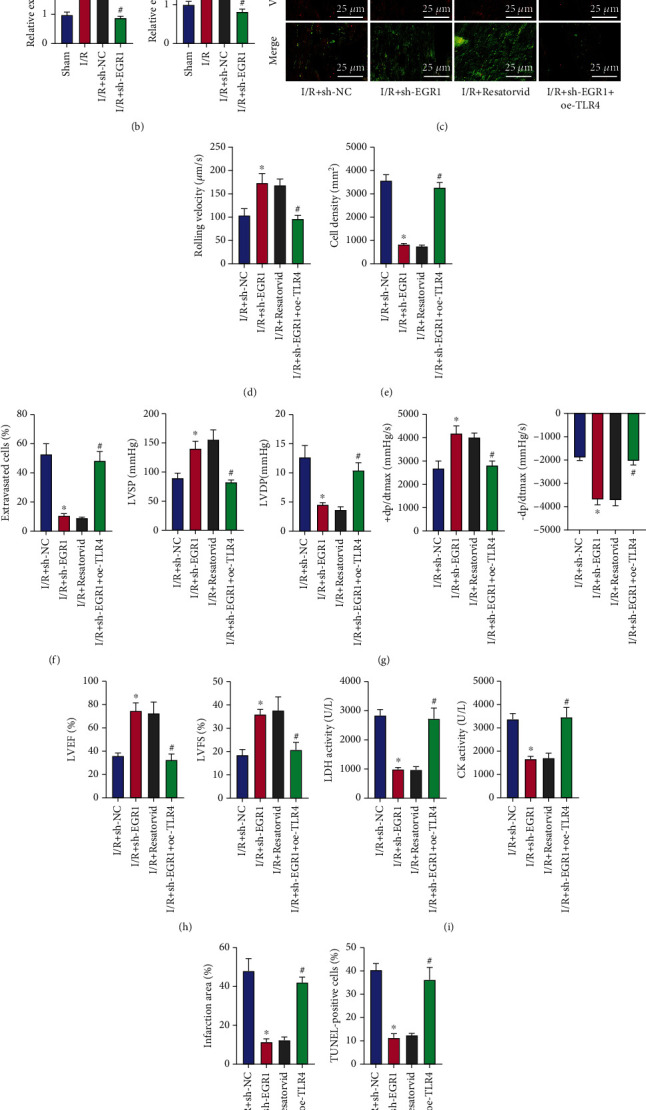
EGR1 silencing diminishes neutrophil recruitment and alleviates I/R injury by inactivating the TLR4/TRIF signaling pathway. (a) Transcriptional binding sites of EGR1 in the TLR4 promoter predicted by the hTFtarget website. (b) Expression of TLR4 and TRIF determined by qRT-PCR in myocardial tissues of sham-operated mice, I/R mice, and sh-EGR1-treated I/R mice. I/R mice were treated with sh-EGR1, resatorvid, and sh-EGR1 + oe-TLR4. (c) Representative microscopic views of neutrophil (green) migration process on the myocardial vascular wall in I/R mice under a two-photon microscope (the vessel was in red). (d) The rolling speed of neutrophils in the blood vessel of I/R mice. (e) The density of neutrophils in I/R mice. (f) The exudation ratio of neutrophils in I/R mice. (g) Detection of LVSP, LVDP, +dp/dtmax, and -dp/dtmax related to cardiac function in I/R mice. (h) LVFS% and LVEF% detected by electrocardiogram in I/R mice. (i) Activity of LDH and CK in the arterial blood of I/R mice. (j) TTC staining of myocardial tissues of I/R mice. (k) Apoptosis of cardiomyocytes in myocardial tissues of I/R mice assessed by TUNEL assay. ^∗^*p* < 0.05 vs. the sham-operated mice or I/R mice treated with sh-NC. #*p* < 0.05 vs. the I/R mice treated with sh-NC or sh-EGR1. *n* = 8 for mice following each treatment.

Next, we sought to analyze the downstream regulatory mechanism of EGR1. Published data have shown that EGR1 often acts as a transcription factor to activate downstream genes in the ischemic arrhythmia and myocardial infarction models induced by oxygen-glucose deprivation/reperfusion injury [18, 19]. In addition, studies have reported that inhibiting EGR1 can reduce the expression of TLR4, and the TLR4/TRIF signaling pathway can aggravate I/R injury by recruiting neutrophils [16, 20]. The hTFtarget website predicted that EGR1 bound to the TLR4 promoter as a transcription factor (Figure 6(a)). Therefore, we speculated that EGR1 may promote the recruitment of neutrophils and thus induce myocardial I/R injury by mediating the TLR4/TRIF signaling pathway.

qRT-PCR data showed higher expression of TLR4 and TRIF in myocardial tissues of I/R mice than that in sham-operated mice. Following EGR1 knockdown, I/R mice had diminished expression of TLR4 and TRIF (Figure 6(b)). In addition, the results of two-photon microscope and chemotaxis experiments showed that in response to treatment with sh-EGR1 or resatorvid, the neutrophils hardly adhered to the blood vessel wall, and the cell rolling speed was accelerated, resulting in a significant decrease in neutrophil density and extravasation and attenuation in neutrophil recruitment. In contrast to treatment with sh-EGR1 alone, combined treatment with sh-EGR1 and oe-TLR4 led to a more pronounced increase in the neutrophil recruitment (Figures 6(c)–6(f)). The above results indicated that knockdown of EGR1 reduced the recruitment of neutrophils by inhibiting TLR4/TRIF signaling pathway.

The results of cardiac function detection and electrocardiogram demonstrated that treatment with sh-EGR1 or resatorvid increased LVSP, +dp/dtmax, -dp/dtmax, LVFS%, and LVEF% while decreasing LVDP. The effects were reverted following combined treatment with sh-EGR1 + oe-TLR4 (Figures 6(g) and 6(h)). In addition, the LDH and CK activity in arterial blood as well as myocardial infarct size and cardiomyocyte apoptosis was attenuated upon EGR1 silencing or TLR4/TRIF inhibition. However, combined treatment with sh-EGR1 and oe-TLR4 could aggravate I/R injury by augmenting the LDH and CK activity in arterial blood and increasing myocardial infarct size and cardiomyocyte apoptosis (Figures 6(i)–(k)).

Overall, these findings supported the promoting effect of EGR1 on the recruitment of neutrophils, and the resultant I/R injury was associated with TLR4/TRIF signaling pathway activation.

## 4. Discussion

Myocardial I/R injury can greatly contribute to tissue injury, which eventually leads to morbidity and mortality in a variety of pathologies, such as myocardial infarction, acute kidney injury. and ischemic stroke [21]. The findings from the present study revealed the mechanism underlying the I/R-induced myocardial tissue injury, where circ_SMG6 initiated neutrophil recruitment and facilitated the ensuing myocardial I/R injury by regulating the miR-138-5p/EGR1/TLR4/TRIF signaling axis.

Our initial results demonstrated the abundant EGR1 expression in myocardial tissues of I/R mice. Additionally, EGR1 silencing inhibited neutrophil recruitment and thereby improved myocardial tissue injury. Consistently, a previous study showed the augmented EGR1 expression in ischemia/hypoxia-exposed cardiomyocytes and that EGR1 suppression protects cardiomyocytes from ischemia/hypoxia-induced injury [22]. Neutrophil recruitment confers an important role in inflammatory process, and following I/R, markedly increased neutrophils are recruited into the myocardium, which contributes to myocardial injury and cardiac dysfunction [23]. The deficiency of EGR1 can decrease neutrophil infiltration in mice treated with transient middle cerebral artery occlusion [24]. Thereby, EGR1 inhibition has the promise to serve as a target for treating myocardial I/R injury by reducing neutrophil recruitment.

Subsequently, miR-138-5p was identified to bind to the 3′UTR of EGR1 mRNA and negatively regulate its expression. This represents the first evidence for posttranscriptional regulation of EGR1 by miR-138-5p in myocardial I/R injury, which may have significance in regulating this disease progression. Furthermore, circ_SMG6 competitively bound to miR-138-5p and caused upregulation of EGR1 expression. Indeed, interaction of circRNA-miRNA-mRNA has been largely reported where circRNAs competitively bind to miRNAs and attenuate their binding ability to the target mRNA expression [25, 26]. The present study also revealed that circ_SMG6 enhanced cardiomyocyte apoptosis and myocardial I/R injury by attenuating miR-138-5p-mediated EGR1 inhibition. Such a circRNA, circ_NCX1 in murine cardiomyocytes and heart tissues promotes the cardiomyocyte apoptosis and I/R injury by functioning as an endogenous sponge of miR-133a-3p [27]. However, circ_SMG6 is novel circRNA and no supporting literature elucidating its role in myocardial I/R injury, let alone in the neutrophil recruitment, and additional studies are thus required for further validation. In addition, miR-138 shows obviously downregulation in H/R-induced cardiomyocytes, while its overexpression impedes H/R injury-induced cardiomyocyte apoptosis and protects cardiomyocytes form H/R injury [28]. A recent study has indicated that low expression of miR-138-5p contributes to the decreased neutrophil accumulation observed in the lipopolysaccharide- (LPS-) induced acute respiratory distress syndrome mice model [29]. This is contrary to our study that miR-138-5p could weaken the neutrophil recruitment, and this discrepancy may be due to the involved study subjects and mechanisms.

Further analysis showed that EGR1 promoted the recruitment of neutrophils and exacerbated I/R injury by activating the TLR4/TRIF signaling pathway. Similarly, suppressed EGR1 expression is capable of disrupting hypoxia-induced TLR4/NF-*κ*B signaling pathway activation, which can facilitate acute myocardial infarction-induced myocardial damage [30]. Meanwhile, the TLR4/TRIF signaling pathway has been established to trigger neutrophil recruitment after heart transplantation and then initiates the progression of I/R injury [16]. These lines of evidence indicated that circ_SMG6 could induce neutrophil recruitment and the subsequent I/R injury via regulation of the miR-138-5p/EGR1/TLR4/TRIF signaling. A relevant point worth mentioning is that, another previously reported mechanism by which activation of the JAK/STAT pathway reduced myocardial I/R injury [31], intersected with our findings, given the participation of STAT1 in TLR signal transduction and inflammatory responses [32]. Moreover, mitochondria confers a crucial role in neutrophils apoptosis [33], and disturbance of mitochondrial homeostasis has been increasingly highlighted in the onset of myocardial I/R injury [34–36]; whether this correlates with the miR-138-5p/EGR1/TLR4/TRIF axis delineated in our study merits future investigations.

**Figure 7:**
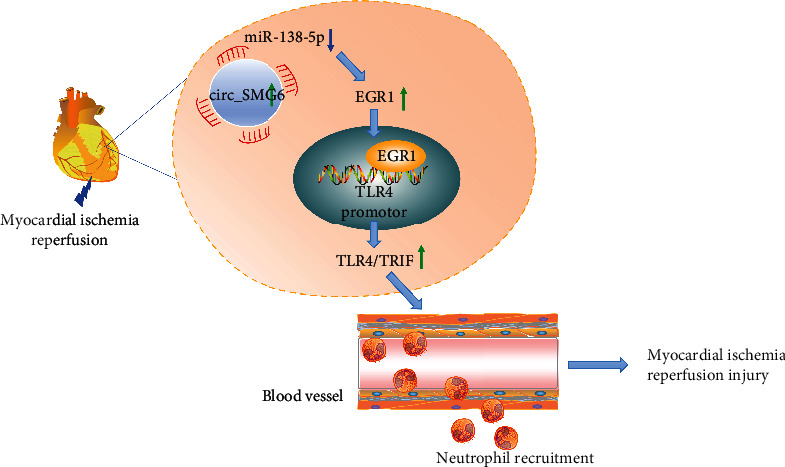
Molecular mechanism underling the circ_SMG6/miR-138-5p/EGR1 regulatory network in myocardial I/R injury. circ_SMG6 and EGR1 are abundantly expressed while miR-138-5p is weakly expressed in myocardial tissues of I/R mice. circ_SMG6 competitively binds to miR-138-5p to upregulate the expression of EGR1 and to promote the binding of EGR1 to the TLR4 promoter, thus activating the TLR4/TRIF signaling pathway. By this mechanism, a large number of neutrophils are recruited, and myocardial I/R injury is ultimately aggravated.

In summary, the findings from the present study suggested that circ_SMG6 promoted neutrophil recruitment and aggravated the resultant I/R injury, which might be related to the downregulation of miR-138-5p, upregulation of EGR1 gene, and TLR4/TRIF signaling pathway activation (Figure 7). Thus, the circ_SMG6/miR-138-5p/EGR1/TLR4/TRIF axis may be potential future therapeutic strategies for myocardial I/R injury. However, further studies are still required for the potential direct role of circ_SMG6 and miR-138-5p in neutrophil recruitment as well as their regulation on the TLR4/TRIF signaling pathway to validate the findings of our study.

## Data Availability

The datasets generated/analyzed during the current study are available.

## Supplementary Materials

Supplementary Materials Supplementary Figure 1: a flow chart of the experimental design in vivo and in vitro. Supplementary Table 1: primer sequences for RT-qPCR. Supplementary Table 2: detailed information of the antibodies used for Western blot analysis. Supplementary Table 3: core degree of each gene in the PPI network.
