# Supplementary material for: Cardioprotective Effect of circ_SMG6 Knockdown against Myocardial Ischemia/Reperfusion Injury Correlates with miR-138-5p-Mediated EGR1/TLR4/TRIF Inactivation
Source: Oxid Med Cell Longev. 2022 Jan 27;2022:1927260. doi: 10.1155/2022/1927260 (PMC8813281; doi:10.1155/2022/1927260)
Supplement: Supplementary Materials — Supplementary Figure 1: a flow chart of the experimental design in vivo and in vitro. Supplementary Table 1: primer sequences for RT-qPCR. Supplementary Table 2: detailed information of the antibodies used for Western blot analysis. Supplementary Table 3: core degree of each gene in the PPI network. [file 1927260.f1.docx]

**Supplementary Table 1** Primer sequences for RT-qPCR

| Gene | Sequence (5’-3’) |  |
| --- | --- | --- |
| circ_SMG6 | Forward: 5’-TGGAAGTACCCGTATGCTGC-3’ | |
|  | Reverse: 5’-CTTTCAACTGGGAGTTATG -3’ | |
| miR-138-5p | Forward: 5’- AGCTGGTGTTGTGAATCAGGCCG -3’ | |
|  | Reverse: Universal reverse primer | |
| EGR1 | Forward: 5’-AGTGATGAACG CAAGAGGCA-3’ | |
|  | Reverse: 5’-GGGAGAAAAGGTCGCTGTCA-3’ | |
| TLR4 | Forward: 5’-AGATCTGAGCTTCAACCCCTTG-3’ | |
|  | Reverse: 5’-GCAGAAACATTCGCCAAGCA-3’ | |
| U6 | Forward: 5’-CTCGCTTCGGCAG CACA-3’ | |
|  | Reverse: Universal reverse primer | |
| GAPDH | Forward: 5’-GGAGAGTGTTTCCTCGTCCC-3’ | |
|  | Reverse: 5’-TTACTCCTTGGAGGCCATGTAG-3’ | |

**Supplementary Table 2** Detailed information of the antibodies used for Western blot analysis

| Antibody | Molecular Weight | Dilution rate | Supplier and Item No. |
| --- | --- | --- | --- |
| Bax | 20kDa | 1:1000 | CST, #89477 |
| Bcl-2 | 26kDa | 1:1000 | CST, #15071 |
| Caspase 3 | 17, 19, 35kDa | 1:1000 | CST, #9662 |
| Cleaved Caspase 3 | 17, 19kDa | 1:1000 | CST, #9664 |
| EGR1 | 75kDa | 1:1000 | CST, #4154 |
| GAPDH | 37kDa | 1:1000 | CST, #2118 |
| Anti-rabbit IgG | | 1:1000 | CST, #7074 |
| Anti-mouse IgG | | 1:1000 | CST, #7076 |

**Supplementary Table 3** Core degree of each gene in the PPI network

| Gene | Degree | Gene | Degree |
| --- | --- | --- | --- |
| MYC | 35 | JAK2 | 27 |
| CD44 | 31 | EGR1 | 26 |
| ICAM1 | 30 | PLEK | 26 |
| RAC2 | 30 | TIMP1 | 26 |
| SELL | 29 | TNFRSF1A | 26 |

Note: Degree represents the number of interactions between the gene and other genes, which is the core degree.


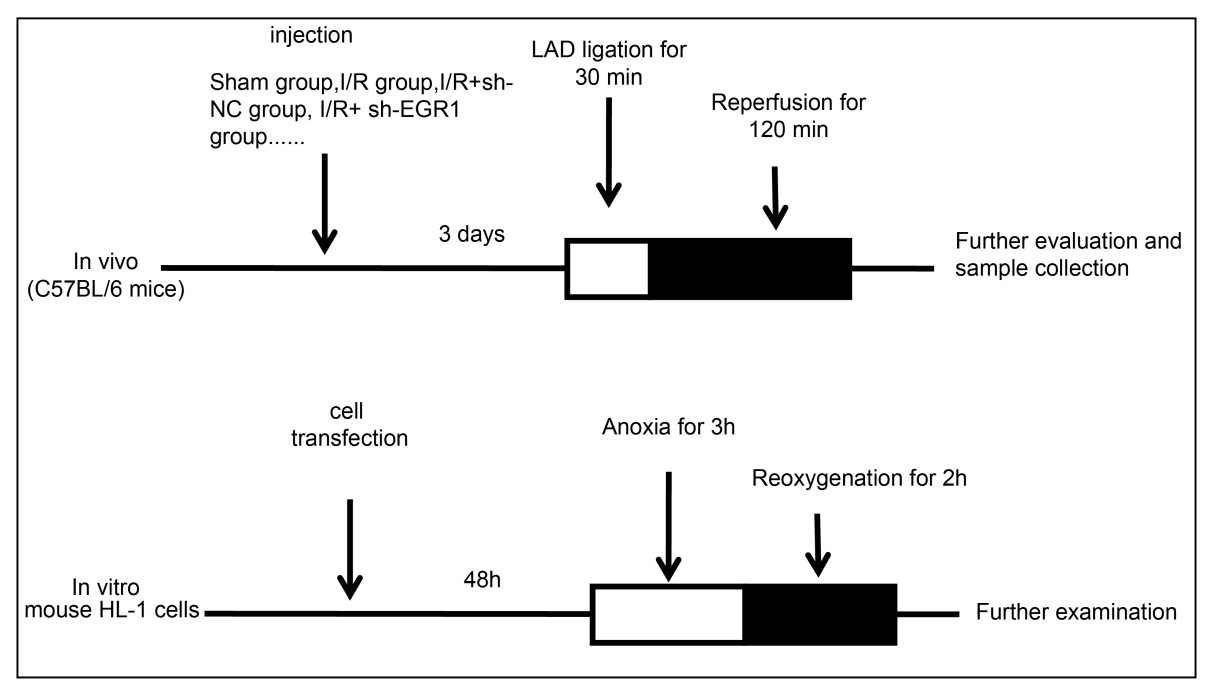


**Supplementary Figure 1** A flow chart of the experimental design *in vivo* and *in vitro.*
